# Endocannabinoid Modulation in Headache: Mechanisms, Models, and Translational Therapies

**DOI:** 10.3390/cells15040331

**Published:** 2026-02-11

**Authors:** Jie Wen, Yumin Zhang

**Affiliations:** Department of Anatomy, Physiology and Genetics, Uniformed Services University of the Health Sciences, 4301 Jones Bridge Road, Bethesda, MD 20814, USA; jie.wen.ctr@usuhs.edu

**Keywords:** endocannabinoid system, trigeminovascular system, descending pain modulation, CGRP signaling, neuroimmune interactions, migraine, traumatic brain injury, sex differences

## Abstract

**Highlights:**

**What are the main findings?**

- The endocannabinoid system (ECS) is a central regulator of neural, vascular, and immune mechanisms driving headache disorders.
- ECS dysfunction contributes to central sensitization and reduced descending inhibition, particularly in chronic headaches.

**What are the implications of the main findings?**

- Targeting endocannabinoid hydrolysis and oxygenation shows consistent preclinical efficacy and represents a promising therapeutic strategy.
- Sex differences significantly influence ECS signaling and therapeutic responsiveness.

**Abstract:**

Headache disorders, including migraine, tension-type headache, trigeminal autonomic cephalalgias, post-traumatic headache and medication overuse headache, represent a major global health burden and remain difficult to treat despite therapeutic advances. The endocannabinoid system (ECS) has emerged as a key regulator of neural, vascular, and immune processes central to headache pathophysiology. Through coordinated actions of CB1 and CB2 receptors, the endogenous ligands anandamide (AEA) and 2-arachidonoylglycerol (2-AG), and their metabolic enzymes, the ECS modulates trigeminovascular activity, descending pain control, cortical excitability, and neuroimmune sensitization. Preclinical studies demonstrate that ECS activation suppresses trigeminal firing, reduces calcitonin gene-related peptide (CGRP) release, attenuates neurogenic inflammation, stabilizes cortical susceptibility to spreading depression, and limits glial activation following traumatic brain injury. Conversely, ECS dysregulation contributes to central sensitization and impaired descending inhibition underlying medication overuse headache and other headache disorders. Pharmacological strategies targeting endocannabinoid degradation, such as inhibition of FAAH, MAGL, and COX-2, enhance endogenous cannabinoid tone and consistently reduce headache-like behaviors across diverse models. Importantly, sex differences shape ECS function, with females exhibiting distinct hormonal regulation, receptor expression, and glial activation that influence responsiveness to ECS-targeted interventions. Collectively, mechanistic and translational evidence highlights the ECS as a promising therapeutic target across primary and secondary headache disorders. Future clinical studies should incorporate sex-informed designs, integrate biomarkers of trigeminovascular and neuroimmune activity, and evaluate peripherally restricted ECS modulators and cannabinoid-based formulations as candidates for individualized headache therapy.

## 1. Introduction

Headache disorders are among the most common and disabling neurological conditions worldwide. Migraine is consistently ranked as a leading cause of disability, particularly among women aged 15–49 years [1], and accounts for nearly 90% of the global disability burden attributed to headache [2]. Tension-type headache is the most prevalent primary headache, affecting about 26% of the population annually [3], whereas cluster headache, though rare at ~0.1% prevalence, leads to severe individual disability [4]. Together, these primary and secondary headache disorders caused by underlying medical conditions produce significant sensory, cognitive, and mood disturbances, reduce productivity, and markedly diminish quality of life.

Despite advances in therapy, unmet clinical needs remain significant. Acute treatments include triptans, ditans, gepants, and nonspecific analgesics, while preventive options encompass beta-blockers, antiepileptic and antidepressant agents, onabotulinumtoxinA, and calcitonin gene-related peptide (CGRP) pathway targeting therapies [5]. The 2024 American Headache Society guidelines designate CGRP-targeted therapies as first-line options for prevention [6]. However, many patients fail to achieve a ≥50% reduction in monthly migraine days. Triptans remain central in acute care but are often contraindicated in patients with cardiovascular risk or advanced age, and inadequate response to triptans is common [7]. Preventive oral therapies are frequently discontinued due to adverse effects or limited perceived benefit, contributing to poor adherence and increasing the risk of progression from episodic to chronic headache [8]. Heavy reliance on acute medications can lead to medication-overuse headache, a secondary disorder driven by maladaptive changes in descending pain modulation and central sensitization [9].

These gaps underscore the need for therapeutic strategies that operate through mechanisms distinct from serotonergic or CGRP-directed pathways. The endocannabinoid system (ECS) is of particular interest because it modulates several processes central to headache pathophysiology, which include trigeminovascular signaling, neuroimmune interactions, and descending pain inhibition. Although migraine is the primary focus, given the depth of available mechanistic and translational evidence, this review also examines other primary headache disorders (tension-type headache and trigeminal autonomic cephalalgias) as well as secondary headache conditions, including post-traumatic headache and medication-overuse headache, where relevant ECS-related data are available. Drug classes are discussed selectively to illustrate underlying mechanisms rather than to provide an exhaustive overview of available therapies. By engaging these convergent mechanisms, ECS-based approaches may complement current treatments and address populations refractory to standard therapies. This review synthesizes current evidence on ECS biology and its therapeutic relevance across headache disorders, with particular emphasis on mechanistic pathways linking endocannabinoid signaling to trigeminovascular activation, neuroimmune sensitization and descending pain modulation, as well as how these mechanisms inform translational and clinical considerations.

## 2. Neurobiological Basis of ECS Modulation in Headache

The ECS consists of the G protein-coupled cannabinoid type 1 (CB1) and type 2 (CB2) receptors, the principal lipid transmitters anandamide (AEA) and 2-arachidonoylglycerol (2-AG), and the enzymes responsible for their synthesis and degradation. AEA is synthesized primarily by N-acyl-phosphatidylethanolamine–phospholipase D (NAPE-PLD) and degraded by fatty acid amide hydrolase (FAAH), whereas 2-AG is produced mainly through diacylglycerol lipase α/β (DAGLα/β) and hydrolyzed by monoacylglycerol lipase (MAGL) [10]. CB1 receptors are highly expressed at presynaptic terminals throughout the central nervous system, where they limit neurotransmitter release and regulate synaptic excitability [11]. In contrast, CB2 receptors are enriched in microglia and peripheral immune cells, positioning them as key modulators of neuroimmune signaling [12]. Through the coordinated actions of these receptors, lipid messengers, and metabolic enzymes, the ECS integrates neuronal and immune processes, making it a central regulator of pathways highly relevant to headache pathophysiology.

To provide a conceptual framework for subsequent sections, Table 1 summarizes the core components of the endocannabinoid system, their localization within headache-relevant circuits, and their functional roles in trigeminovascular signaling, descending pain modulation, and neuroimmune regulation.

### 2.1. Integrated ECS Regulation of Trigeminovascular Signaling and Descending Pain Control

The trigeminovascular system and descending pain modulatory circuits form a functionally coupled network that governs headache initiation and persistence. Accumulating evidence indicates that the ECS regulates this network at multiple levels, coordinating peripheral trigeminal input, brainstem processing, and top–down inhibitory control. At the meningeal and trigeminal levels, ECS signaling modulates CGRP release and trigeminovascular excitability. Recent ex vivo and anatomical evidence indicates that CB1 receptors are predominantly localized to trigeminal ganglion (TG) neuronal somata and Aδ-fibers, where they colocalize with CGRP and receptor activity-modifying protein 1 (RAMP1), whereas CB2 receptors are mainly expressed in satellite glial cells with minimal colocalization with CGRP-positive neurons. Under basal conditions, CB1 agonism alone has limited inhibitory effects on CGRP release. However, when TRPV1 activity is suppressed, CB1-dependent inhibition of CGRP release becomes evident, highlighting a state-dependent and indirect role for CB1 signaling in trigeminovascular modulation [28]. Activation of the transient receptor potential vanilloid 1 (TRPV1) and ankyrin 1 (TRPA1) ion channels drives CGRP release and meningeal vasodilation, key contributors to migraine pain [26,27]. AEA exerts state-dependent effects within this axis: it activates TRPV1 to promote CGRP-mediated vascular responses, while CB1 receptor activation suppresses neurogenic dural vasodilation and CGRP release [13,17]. Ex vivo studies demonstrate that CB1-mediated suppression of CGRP release becomes evident only when TRPV1 activity is inhibited, indicating that TRPV1 signaling can mask cannabinoid-dependent inhibition under conditions of heightened excitability [28].

Within the brainstem, ECS signaling suppresses trigeminovascular transmission and engages in descending inhibitory pathways. CB1 activation reduces Aδ- and C fiber-evoked firing of second-order neurons in the trigeminocervical complex (TCC) [13] and attenuates dural-evoked responses within the ventrolateral periaqueductal gray (vlPAG), consistent with recruitment of PAG–rostral ventromedial medulla (RVM) inhibitory circuits [31]. In the PAG, activity-dependent synthesis of AEA and 2-AG activates presynaptic CB1 receptors to suppress GABA and glutamate release, shifting RVM circuitry toward OFF cell-mediated inhibition and limiting ON cell-driven facilitation [11,32,33,34]. Through this dual synaptic control, ECS signaling enhances antinociceptive output from the PAG–RVM–TCC axis and functionally parallels opioid-mediated analgesia [16,19]. Importantly, ECS actions within brainstem regions such as the PAG and RVM occur behind the blood–brain barrier (BBB). In contrast, TG neurons and associated satellite glial cells lie outside the BBB, allowing direct access to circulating mediators and peripherally restricted pharmacological agents [35,36].

**Figure 1:**
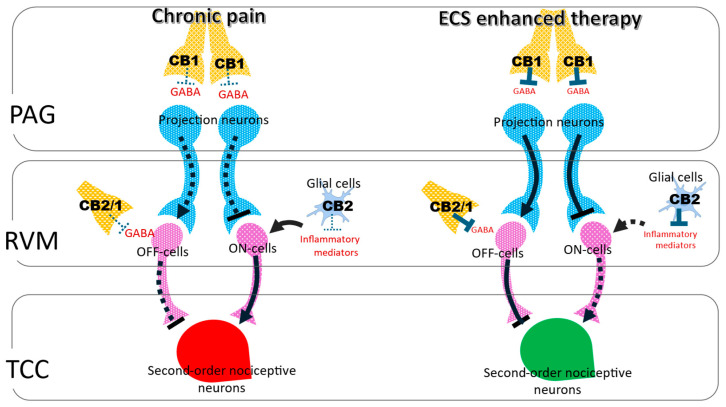
Endocannabinoid-enhanced regulation of the descending pain modulatory pathway. Left side (chronic pain state): Under chronic pain conditions, nociceptive input from trigeminal afferents is transmitted to second-order nociceptive neurons within the trigeminocervical complex (TCC; shown in red) and modulated by descending projections from the periaqueductal gray (PAG) to the rostral ventromedial medulla (RVM). Descending projection neurons within the PAG–RVM axis (shown in blue) regulate RVM circuitry, where pain-facilitating ON-cells and pain-inhibitory OFF-cells (shown in purple) exert bidirectional control over trigeminovascular nociceptive transmission. CB1 and CB2 receptors expressed within the PAG and RVM (shown in yellow) are present but insufficiently engaged due to reduced or dysregulated endocannabinoid tone. Consequently, insufficient CB1 signaling allows persistent GABAergic inhibition of PAG projection neurons, reducing RVM OFF-cell activity and favoring ON-cell–mediated facilitation. This imbalance permits sustained excitation of second-order nociceptive neurons in the TCC, promoting central sensitization and persistent headache pain. Right side (ECS-enhanced descending inhibition): Pharmacological enhancement of endocannabinoid signaling strengthens descending pain control. Inhibition of endocannabinoid hydrolytic and metabolic enzymes FAAH, MAGL, or COX-2 via substrate-selective inhibition elevates AEA and 2-AG levels. Enhanced activation of CB1 and CB2 receptors within the PAG and RVM reduces excessive GABAergic inhibition of descending projection neurons, thereby increasing descending output to the RVM. This enhanced ECS signaling shifts RVM activity toward OFF-cell activation and suppression of ON-cell firing, resulting in effective inhibition of second-order nociceptive neurons within the TCC. In parallel, CB2 receptor activation on microglia and immune cells attenuates neuroinflammatory signaling. Together, coordinated CB1- and CB2-mediated actions restore descending inhibitory tone and highlight ECS enhancement as a therapeutic strategy for chronic headache and post-traumatic headache disorders.

In chronic headache and persistent pain states, reduced endocannabinoid tone weakens both trigeminovascular and descending inhibitory control. Insufficient CB1 engagement permits sustained CGRP signaling, elevated trigeminovascular gain, and diminished recruitment of RVM OFF cells. Under these conditions, CB2 receptors become increasingly relevant due to their upregulation in microglia and immune cells, which dampens neuroinflammatory signaling and limits central sensitization [11,16,19]. Pharmacological strategies that enhance endogenous cannabinoid tone via FAAH or MAGL inhibition restore CB1-dependent suppression of trigeminovascular transmission while engaging CB2-mediated immunomodulation, thereby strengthening integrated control across peripheral and central headache circuits [34]. To integrate these mechanisms within a unified framework, the organization of the descending pain modulatory system and its likely regulation by endocannabinoid signaling are summarized in Figure 1.

### 2.2. Endocannabinoid System Regulation of Neuroimmune Signaling and Neural Excitability Homeostasis

While ECS regulation of trigeminovascular signaling and descending pain pathways provides control of headache processing at the circuit level, these effects are sustained and amplified by parallel actions on neuroimmune signaling and cellular excitability. At this level, the ECS functions as a homeostatic system that limits inflammation-driven sensitization and stabilizes the hyperexcitable neural network.

Activation of CB2 receptors expressed on microglia, perivascular macrophages, and select astrocyte populations attenuates neuroimmune signaling by suppressing NF-κB and MAPK pathways, reducing pro-inflammatory cytokine release, and promoting anti-inflammatory mediators such as IL-10 [12,37]. In headache-relevant models, CB2 agonism or enhancement of endocannabinoid tone reduces microglial activation, limits central sensitization, and reverses cephalic allodynia [15]. At the meningeal interface, CB2 signaling also regulates mast cell and macrophage activity, thereby dampening neurogenic inflammation and CGRP release [38]. Together, these mechanisms position CB2-mediated immunomodulation as a critical counterbalance to the chronic inflammatory state observed in post-traumatic headache and persistent migraine.

In parallel, the ECS contributes directly to the homeostatic regulation of neuronal excitability. Endocannabinoids are synthesized on demand and act locally to stabilize hyperexcitable circuits by limiting excessive synaptic transmission [10]. Augmentation of endocannabinoid tone through inhibition of hydrolysis or oxygenation enhances these adaptive responses while avoiding the psychoactive liabilities associated with direct CB1 agonists [18,29]. ECS signaling intersects multiple headache-relevant pathways, including CGRP signaling, cortical spreading depression, and meningeal neuroinflammation [39]. CB1 receptors are abundantly expressed throughout the PAG, RVM, TCC, and cerebral cortex, where interactions with serotonergic systems and TRP channels further shape sensory processing and neurovascular responses [32,40]. Consistent with this integrative role, cannabinoid ligands suppress neuropeptide release and neurogenic dural vasodilation across experimental preparations [13].

Collectively, ECS-mediated neuroimmune regulation and excitability control reinforce the inhibitory actions described at the trigeminovascular and descending circuit levels. By coordinating CB1- and CB2-dependent mechanisms across neurons, glia, and immune cells, the ECS increases the threshold for headache initiation, limits the propagation of nociceptive signals, and constrains the transition from episodic to chronic pain states. The following section examines how these integrated cellular, circuit, and immune mechanisms are interrogated in experimental and preclinical headache models, which provide critical insight into how ECS modulation translates into measurable behavioral, electrophysiological, and neurochemical outcomes.

## 3. Experimental and Preclinical Studies of ECS Modulation in Headache Models

Building on mechanistic insights into ECS regulation of pain, the next step is to examine how these pathways are engaged in experimental and preclinical models of headache. Such models encompass a wide range of paradigms, from trigeminovascular and meningeal preparations to nitroglycerin-induced migraine, cortical spreading depression and aura-linked paradigms, dural challenge models, trigeminal autonomic cephalalgia, post-traumatic headache platforms, and medication overuse headache. Together, they provide complementary perspectives on how the ECS shapes neuronal excitability, vascular reactivity, neuropeptide release and neuroimmune signaling in headache biology. These studies pave a foundation for translating ECS modulation into rational therapeutic strategies. The following sections summarize key findings across these diverse models, highlighting both shared mechanisms and disorder-specific insights.

### 3.1. Experimental Evidence of ECS Regulation in Trigeminovascular and Meningeal Models

Experimental paradigms using trigeminovascular and meningeal preparations provide the initial evidence that the ECS participates in headache biology. These models permit direct assessment of how cannabinoid receptor activation influences nociceptive processing in the TCC and vascular responses at the meningeal interface, which are two core components of migraine pathophysiology.

Extracellular electrophysiological studies provide direct evidence that cannabinoid signaling modulates trigeminovascular and meningeal activity. Recordings from the TCC show that CB1 agonists suppress Aδ- and C-fiber-driven neuronal firing, whereas CB1 antagonists enhance trigeminal responses, demonstrating a bidirectional influence of CB1 signaling on trigeminovascular gain relevant to migraine and other primary headaches [14]. Using similar approaches, CB1 activation in the vlPAG reduces dural-evoked Aδ fibers firing and decreases spontaneous TCC activity, effects that are reversed by CB1 antagonists, supporting a role for endogenous cannabinoids in brainstem modulation of trigeminovascular output [31]. Complementary experimental studies at the meningeal level show that activation of TRPV1 and TRPA1 reliably induces CGRP release and meningeal vasodilation, and that administration of AEA alters these CGRP-dependent vascular and nociceptive readouts across in vivo and ex vivo preparations [13,17,26,27,41], supporting the ECS as a functional modulator of trigeminovascular neuronal activity, neuropeptide release, and meningeal vascular responses. Consistent with this, ex vivo hemiskull and trigeminal tissue studies show that the CB1 agonist ACEA inhibits K^+^- or capsaicin- evoked CGRP release only when TRPV1 is blocked, demonstrating that TRPV1 activity can mask cannabinoid-mediated analgesia [28,42,43,44]. Within TG, CGRP serves as a marker of neuronal activation. However, recent evidence indicates that ECS modulation exerts only weak effects on CGRP-positive C-fiber neurons with minimal direct involvement of CB2 receptors on neurons. Instead, CB2 signaling in the TG appears to act predominantly through satellite glial cells, supporting an indirect, neuroimmune mechanism of ECS action at the peripheral trigeminal level [28].

A complementary model preserving the intact TCC–TG circuit shows that TNC stimulation alone evokes peripheral CGRP release even without direct meningeal input [45], demonstrating the retrograde influence of central neurons on primary afferents. Dopamine increases TNC-driven CGRP release from the TG, and this effect is prevented by blocking TRPV1 or inhibiting NAPE-PLD, suggesting that dopamine can enhance the synthesis of AEA, which acts through TRPV1 to control CGRP release [28]. Although both AEA and 2-AG suppress nociceptive firing in meningeal afferents, AEA seems to produce a more sustained effect, partially due to the higher FAAH activity compared to the MAGL in meningeal tissues [23,46,47,48,49]. Therefore, the balance between CB1-mediated inhibition and TRPV1-driven excitation determines the net outcome of AEA signaling. When TRPV1 is engaged, AEA signaling is shifted toward excitatory pathways, weakening CB1-mediated suppression. By contrast, FAAH inhibition prolongs AEA availability and promotes preferential CB1 signaling, enhancing analgesia. These findings highlight CB1–TRPV1 crosstalk as a key regulatory bottleneck. Therefore, combining peripherally biased FAAH inhibition to enhance local AEA tone with TRPV1 modulation may improve the reliability of CB1-mediated suppression of trigeminovascular activity while minimizing TRPV1-dependent pro-nociceptive responses.

### 3.2. Studies on ECS Modulation in the Nitroglycerin-Induced Migraine Model

Electrophysiological and meningeal vascular studies first established that endocannabinoids regulate trigeminovascular signaling, providing a foundation for testing ECS modulation in whole-animal migraine models. Systemic administration of nitroglycerin (NTG) is one of the most widely used preclinical migraine paradigms, as it links circuit-level alterations to behavioral and molecular readouts. NTG reliably induces cephalic allodynia, photophobia-like behavior, and facial grimace, accompanied by increased CGRP release, activation of c-Fos and pERK in the TCC, and elevated pro-inflammatory cytokine production. Importantly, many of these outcomes can be assessed using repeated behavioral testing and serial blood sampling, allowing longitudinal tracking of behavioral sensitization and circulating biomarkers, including CGRP and cytokines, within the same animals over time [50,51,52,53]. In contrast, tissue-based measures of CGRP, nNOS, and c-Fos require terminal collection and are therefore obtained from staggered cohorts at defined post-NTG time points to capture temporal dynamics. These endpoint analyses are complemented by in vivo techniques such as microdialysis, fiber photometry, and calcium imaging, which enable real-time monitoring of neurochemical release and neuronal activity in freely moving animals [21,22,54,55].

Both ex vivo hemiskull preparations and in vivo animal migraine models demonstrate that CB1 activation suppresses trigeminovascular output and neuropeptide release. Methanandamide suppresses NTG-evoked CGRP release from plasma, TG, brainstem, and meninges. This suppression is blocked by CB1 antagonists but not by TRPV1 or CB2 antagonists except in mast cells [43]. These findings indicate that activation of CB1 dampens NTG-induced neurogenic inflammation.

Peripherally restricted FAAH inhibition with URB937 reduces both acute and chronic NTG-induced hypersensitivity, decreases CGRP and substance P release, and lowers the production of pro-inflammatory cytokines in the TG and brainstem. These effects are blocked by the CB1 antagonists, demonstrating a CB1-dependent mechanism and supporting peripheral ECS augmentation as a therapeutic strategy [21,22]. Similarly, the global FAAH inhibitors, such as ARN14633 and ARN14280, reverse NTG-induced pain-like behaviors and downregulate the production of CGRP, nitric oxide, TNF-α, IL-1β, and IL-6 through enhancement of the endocannabinoid tone [23,55].

Dual FAAH/MAGL inhibition with JZL195 is also shown to produce robust antinociceptive effects. It reduces NTG-induced orofacial hyperalgesia, serum CGRP, and pro-inflammatory transcripts in the TG and cervical spinal cord. These effects are abolished by the CB1 antagonists, reinforcing the CB1-dependent mechanisms [54,56]. Selective MAGL inhibition alone is also able to alleviate NTG-induced periorbital allodynia, suggesting the important role of preserving 2-AG signaling in mitigating migraine-like pain [24,57].

In addition to behavioral and neuropeptidergic outcomes, the NTG model shows that AEA modulates multiple migraine-relevant pathways beyond canonical cannabinoid receptor signaling. In NTG-treated animals, AEA attenuates trigeminal sensitization by reducing TRPV1 activity and suppressing the expression of neuronal nitric oxide synthase (nNOS), NF-κB and COX-2 in the C1 and C2 dorsal horns, thereby limiting inflammation-mediated central sensitization [46]. AEA further suppresses NTG-induced increase in the serotonin transporter (5-HTT), suggesting that endocannabinoids can modulate headache by interacting with the serotonergic pathways [58]. Very recently, the same research group showed that AEA can normalize the expression of Ca^2+^/calmodulin-dependent protein kinase II (CaMKII) and vasoactive intestinal polypeptide type 1 receptor (VPAC1) [59], which is consistent with its modulatory role on excitatory plasticity and neurogenic inflammation induced by other neurogenic peptides—pituitary adenylate cyclase-activating polypeptide (PACAP) and vasoactive intestinal polypeptide (VIP)—that trigger the onset of migraine in experimental animals and migraineurs [60].

It has also been demonstrated that NTG-induced migraine-like states engage in the kynurenine pathway, a key regulator of glutamatergic transmission and neuroimmune signaling. NTG shifts tryptophan metabolism toward kynurenine derivatives that promote NMDA receptor–dependent excitatory signaling and central sensitization. Importantly, endocannabinoid signaling functionally intersects with the kynurenine pathway through AEA-modulated NTG-induced alterations in kynurenine metabolism, thereby influencing glutamatergic excitability and sensitization [61]. Beyond excitatory neurotransmission, kynurenines play a critical role in migraine-related neuroimmune pathways by regulating microglial activation, cytokine release and oxidative stress within trigeminovascular circuits. These immune-modulatory actions converge with established endocannabinoid functions at the neuroimmune interface, suggesting that interactions between ECS and kynurenine represent a shared point of convergence linking NTG-induced neuroinflammation and neuronal sensitization [62]. Collectively, these findings establish the NTG model as an integrative framework linking ECS modulation to excitatory, inflammatory, and neuroimmune mechanisms underlying migraine.

### 3.3. ECS Modulation in Cortical Spreading Depression and Aura-Linked Migraine Models

Findings from NTG-induced migraine models demonstrate that ECS modulation alleviates migraine-like pain and normalizes CGRP signaling in vivo. Migraines also involve cortical dysfunction, which is mostly expressed as a visual or sensory aura. Cortical spreading depression (CSD), a propagating wave of neuronal and glial depolarization, is the recognized substrate of aura and a trigger of downstream trigeminovascular activation [63,64,65]. By lowering cortical excitation thresholds, CSD produces transient neurological symptoms and primes nociceptive pathways for headache onset. Given the ECS’s modulatory influence on synaptic transmission and balance between excitation and inhibition, its ability to regulate CSD dynamics has emerged as a promising therapeutic target.

Experimental studies show that CB1 receptor activation in rat neocortical slices reduces the incidence and propagation of CSD, indicating that cannabinoid signaling raises the threshold for aura initiation [66]. Enhancing endogenous ECS tone produces similar effects. The multi-target hydrolase inhibitor AKU-005, which blocks MAGL, FAAH and ABHD6, elevates cortical AEA and 2-AG levels and reduces CSD frequency [67]. Substrate-selective COX-2 inhibitors (SSCIs) also enhance endocannabinoid signaling and improve headache-relevant outcomes, such as reducing pain behaviors and neuroinflammation in post-traumatic headache models [29,30].

In a KCl-evoked CSD animal model, the expression of MAGL and ABHD6 was increased in the PAG, and inhibition of either enzyme prevented or reversed periorbital allodynia, suggesting that augmentation of the endogenous levels of 2-AG can mitigate aura-associated headache [20]. Like the selective MAGL inhibitors, broad inhibition of endocannabinoid hydrolysis with AKU-005 also decreased CSD and stabilized cortical excitability in a CB1-dependent manner [57,68].

The role of TRP channels is less clearly defined. TRPV1 and TRPA1 can modulate cortical excitability and may influence the coupling between aura and headache. However, direct TRPV1 blockade has not been consistently shown to suppress CSD [66,67]. Together, these findings indicate that direct TRPV1 inhibition alone is insufficient to consistently suppress CSD, whereas indirect modulation of TRP channel activity, potentially via endocannabinoid signaling, may provide a more effective means of regulating cortical excitability and the coupling between aura and headache.

Clinical and translational findings support the relevance of CSD to migraine with aura. Neuroimaging and electrophysiological studies demonstrate that CSD aligns with the spatiotemporal dynamics of migraine aura symptoms [64,69], and both human and animal experimental evidence supports that CSD triggers downstream trigeminovascular activation [63,65]. Although ECS-targeted therapies have not yet been evaluated clinically for aura, preclinical data implicate their potential to stabilize cortical excitability and reduce susceptibility to CSD.

### 3.4. Role of the Endocannabinoid System in Dural Challenge Models

CSD- and aura-linked paradigms illustrate how the ECS stabilizes cortical excitability, whereas headache pain following aura is driven largely by peripheral meningeal mechanisms. To investigate these pathways, researchers employ dural challenge models including inflammatory soup (IS), TRP agonists such as capsaicin or AITC, and non-invasive dural stimulation, which enable direct assessment of how ECS modulation influences meningeal CGRP release, vascular reactivity and trigeminovascular activation in both in vivo and ex vivo settings.

IS, a mixture of bradykinin, histamine, serotonin, and prostaglandin E2, reliably activates and sensitizes trigeminal afferents, increasing CGRP, TRPV1 and pERK1/2 expression and producing sustained allodynia that mimics chronic headache states [66]. Although ECS-directed studies in IS models are limited, findings from related meningeal paradigms show that peripherally restricted FAAH inhibition with URB937 reduces trigeminal activation and downregulates CGRP-related transcription, suggesting similar efficacy in IS-induced sensitization [21].

TRP-driven models provide further mechanistic insight. Capsaicin (TRPV1 agonist) and AITC (TRPA1 agonist) selectively activate meningeal afferents and evoke robust CGRP release and vascular dilation. AITC produces consistent dural and pial artery dilation, offering a reproducible vascular readout of migraine-like signaling [70]. Endocannabinoids such as AEA and NADA increase meningeal blood flow and stimulate CGRP release through TRPV1 activation, and these effects are prevented by TRPV1 antagonists or CGRP receptor blockers [13,71]. In contrast, CB1 activation suppresses trigeminovascular excitability and reduces CGRP secretion. When CB1 receptors are blocked, AEA-induced vasodilation becomes more pronounced, highlighting a functional interplay between CB1 and TRPV1 signaling. More recent work shows that CB1 agonists inhibit CGRP release only when TRPV1 is concurrently suppressed, highlighting state-dependent CB1-TRPV1 crosstalk [28]. Collectively, TRP-based models provide strong evidence that ECS modulation influences vascular and neuropeptide pathways central to migraine.

Non-invasive dural stimulation (NIDS) adds a complementary approach through electrically activating the dura without craniotomy. NIDS induces migraine-like hypersensitivity and neuroinflammation and is well-suited for longitudinal pharmacological studies [72]. Although ECS-targeted interventions remain largely unexplored in NIDS, the model is ideal for testing peripherally restricted FAAH and MAGL inhibitors and CB1 receptor-biased ligands with respect to CGRP release, cytokine expression, and facial hypersensitivity.

Together, IS, TRP-driven, and NIDS models provide consistent evidence that the ECS regulates meningeal excitability, vascular tone, and neuropeptide release. These preclinical findings align with human data demonstrating that TRP channel activation and CGRP release are key drivers of migraine pathology. Accordingly, ECS-directed interventions, including peripherally restricted FAAH inhibition (URB937), dual FAAH/MAGL inhibition (JZL195) and CB1 receptor agonism, reduce trigeminal activation and suppress CGRP output across multiple models [28,39]. By integrating vascular, molecular and behavioral endpoints, dural challenge models offer a clinically relevant framework for evaluating ECS-based therapies and reinforcing the rationale for targeting ECS in headache disorders.

### 3.5. ECS in Trigeminal Autonomic Cephalalgias (TAC)–Oriented Model

TACs are severe primary headaches characterized by unilateral pain with prominent cranial autonomic features such as tearing and nasal congestion [73]. Mechanistic research was historically limited by the absence of validated models. TAC paradigms address this gap by activating the trigeminal–autonomic reflex through stimulation of brainstem or hypothalamic circuits, most commonly the superior salivatory nucleus. This approach produces single-unit firing in the TCC accompanied by lacrimation, nasal secretion, and cranial blood flow changes measured via extracellular recordings and laser Doppler flowmetry [74,75]. These readouts closely parallel clinical TAC features and provide a robust framework for mechanistic and pharmacological testing.

Established TAC therapies, including high-flow oxygen, indomethacin and triptans, reliably suppress reflex-evoked responses in these models, whereas CGRP antagonists show more limited efficacy. This pharmacological profile reinforces the translational validity of TAC paradigms and supports their use in evaluating emerging approaches that include the ECS modulation. Within these models, ECS modulation interacts with TAC circuitry functionally in relevant ways. CB1 activation suppresses TCC responses to dural Aδ- and C-fiber input and reduces dural-evoked activity in the vlPAG; these effects are reversed by CB1, but not CB2, antagonism [14]. By integrating neural and autonomic vascular outputs, TAC models allow detailed analysis of how ECS modulation affects the trigeminal–autonomic reflex, offering broader insight than the migraine-specific paradigms. These characteristics make TAC paradigms especially valuable for early ECS drug development. They not only distinguish TAC-effective agents such as oxygen, indomethacin, and triptans from migraine-selective therapies, but also allow careful evaluation of ECS strategies such as peripherally biased FAAH or MAGL inhibitors and CB2 agonists. The ability to compare reflex-driven versus direct dural activation further distinguishes CB1-mediated analgesia from nonspecific sedation, thereby strengthening translational confidence.

Clinically, evidence for cannabinoid effects in TACs remains limited. Observational studies in cluster headache reveal mixed responses to cannabis, ranging from reported relief to no benefit or symptom worsening [76]. No randomized controlled trials have yet evaluated ECS-targeted therapies in TAC patients.

Overall, TAC models reproduce both pain and autonomic components of the disorder, aligning with known clinical pharmacology, providing an accessible platform for ECS testing. Current preclinical evidence shows that CB1 activation suppresses trigeminal activity and modulates descending control, while clinical data underscore the need for controlled trials. Testing ECS interventions in TAC models should be followed by a targeted clinical evaluation. Using endpoints such as attack frequency, autonomic signs, and acute medication use will help advance ECS-based therapies beyond migraine and post-traumatic headache.

### 3.6. Endocannabinoid Modulation in Experimental Models Relevant to Tension-Type Headache

Unlike migraine, tension-type headache (TTH) does not have a single pathognomonic experimental model; instead, its pathophysiology is investigated using validated paradigms that reproduce its defining mechanisms, including sustained muscle nociception, stress-induced hyperalgesia, impaired descending pain inhibition, and central sensitization. Persistent muscle pain models, such as repeated intramuscular acid or hypertonic saline injections, produce long-lasting hypersensitivity and referred pain that closely mirror pericranial muscle tenderness and central amplification observed in chronic TTH [77,78]. These paradigms reliably produce bilateral pain, receptive field expansion, and a progressive loss of endogenous inhibitory control, closely recapitulating key features of chronic TTH. In parallel, stress-based paradigms, including repeated restraint or chronic mild stress, impair descending inhibitory control arising from the PAG–RVM axis, a core abnormality documented in TTH patients [79,80].

Across these models, endocannabinoid signaling plays a central regulatory role. CB1 receptor activity within the PAG–RVM circuit is essential for maintaining tonic descending inhibition, and pharmacological enhancement of endocannabinoid tone via FAAH or MAGL inhibition restores antinociceptive control and reduces muscle- and stress-evoked hyperalgesia in a CB1-dependent manner [18,32,33,34]. In addition, CB2-mediated modulation of glial and immune activity attenuates neuroimmune contributions to central sensitization in chronic pain states relevant to TTH [19,81].

Translational support for these experimental findings is provided by recent clinical biomarker studies. An observational study using salivary lipid profiling demonstrated that patients with chronic TTH exhibit altered endocannabinoid signatures, with particular dysregulation of 2-AG levels, suggesting impaired endocannabinoid-mediated pain control in this population [82]. This non-invasive assessment supports the concept that deficient or maladaptive 2-AG signaling contributes to sustained nociceptive gain and impaired descending inhibition in TTH, aligning with preclinical evidence implicating MAGL-regulated 2-AG tone in chronic pain modulation.

### 3.7. ECS Modulation of Neuroimmune Mechanisms in Post-Traumatic Headache

Dural inflammatory soup, TRP agonist (capsaicin/AITC), and NIDS models illustrate how the endocannabinoid system regulates meningeal CGRP release, vascular tone, and peripheral nociceptor activity at the trigeminovascular interface. Building on these peripheral mechanisms, TAC and TTH models extend ECS regulation to central brainstem circuits, autonomic reflexes, impaired descending pain inhibition, and stress- and muscle-driven sensitization that characterize primary headache disorders. In contrast to these primary headache disorders, post-traumatic headache (PTH) is a secondary headache in which traumatic brain injury (TBI) triggers persistent neuroimmune activation, central sensitization, and maladaptive trigeminovascular signaling, all of which are the pathological processes that are strongly modulated by endocannabinoid system function. Accordingly, experimental platforms including the closed-head impact model of engineered rotational acceleration (CHIMERA), weight-drop, controlled cortical impact (CCI), fluid percussion injury (FPI), and blast exposure consistently demonstrate that ECS modulation attenuates neuroinflammation, suppresses glial reactivity, and reduces headache-like behaviors [83].

PTH is a common and disabling consequence of TBI, and its persistence is driven by trigeminovascular sensitization and sustained neuroimmune activation. Among existing models, the CHIMERA closed-head injury paradigm offers the most compelling evidence for ECS involvement. Inhibition of 2-AG hydrolysis with selective MAGL inhibitors reduces periorbital allodynia, decreases CGRP expression in trigeminal pathways, and suppresses microglial and astrocytic activation [25]. Substrate-selective COX-2 inhibition with IMMA similarly preserves endocannabinoid tone, limits neuroinflammation and mast cell activation, and ameliorates headache-like behaviors [30]. FAAH inhibition with PF-04457845 is also found to improve functional recovery and reduce neuroinflammation in the closed-head injury mouse model [84]. These findings demonstrate that strengthening AEA or 2-AG signaling can mitigate behavioral and molecular features of PTH.

Weight-drop models further support this framework. Diffuse injury in these paradigms produces persistent cephalic allodynia and heightened sensitivity to normally innocuous triggers such as CGRP or NTG weeks after injury [85,86,87]. Although ECS-directed interventions have not been extensively tested in these models compared to CHIMERA, they nonetheless provide a robust behavioral platform to evaluate FAAH and MAGL inhibitors in reducing post-injury hypersensitivity.

Focal injury models such as CCI reveal dysregulation of the ECS after TBI. CCI is associated with reduced cortical AEA and related fatty acid ethanolamides together with persistent allodynia and glial activation [88]. Subsequent work has demonstrated that these reductions are accompanied by upregulation of endocannabinoid-degrading enzymes, including FAAH and MAGL, contributing to sustained ECS imbalance after injury [89]. Inhibition of MAGL restores 2-AG levels, reduces post-injury pain behaviors and dampens glial activity, supporting a therapeutic role for controlling the 2-AG metabolism [84,90].

The FPI model, which incorporates both diffuse and focal injury components, shows that FAAH and MAGL inhibition improves cortical excitability, synaptic plasticity and inflammatory profiles, although headache-specific endpoints are less commonly evaluated [91]. However, these results indicate that ECS augmentation mitigates TBI-induced physiological disruptions linked to headache biology.

Blast injury models also add complementary evidence. Following focal blast exposure, CB2-biased intervention with SMM-189 improved motor, visual, and affective outcomes and promoted reparative microglial phenotypes [92]. Although headache-related measures were not assessed, these studies highlight ECS-mediated immunomodulation as a potential therapeutic mechanism in blast-related TBI.

Clinical translation of ECS interventions remains limited, but key milestones demonstrate feasibility. Positron emission tomography (PET) imaging confirms central FAAH occupancy with PF-04457845 [93]. Observational reports indicate that increasing patient use of cannabinoid-based formulations including cannabidiol (CBD) helps to manage headache, sleep, and mood symptoms following TBI [94]. These findings underscore the need for controlled clinical trials aimed specifically at PTH.

Across CHIMERA, weight-drop, CCI, FPI, and blast models, ECS modulation consistently reduces headache-like behaviors, suppresses CGRP expression, and attenuates neuroimmune activation. This convergence validates the ECS as a therapeutic target in PTH and supports further development of peripherally biased FAAH/MAGL inhibitors, SSCIs, CB2 agonists, and CBD-based formulations for future clinical evaluation.

### 3.8. Therapeutic Potential of ECS Modulation in Medication Overuse Headache

Medication overuse headache (MOH) is the most common secondary headache disorder, affecting roughly 1–2% of the general population and up to 50% of patients seen in specialized headache clinics [9]. MOH develops in susceptible individuals with primary headache disorders such as migraine or TTH who overuse acute medications such as triptans, NSAIDs, simple analgesics, combination products, or opioids [95]. The pathophysiology of MOH involves maladaptive changes in pain processing networks, including altered descending modulation, enhanced central sensitization, and neuroimmune activation [96]. Given the regulatory role of ECS on trigeminovascular activity, neuroimmune signaling and descending pain modulation, it is reasonable to believe that ECS modulation can be effective in the treatment of MOH.

Most animal models of MOH rely on repeated administration of triptans or opioids to mimic the transition from episodic to chronic headache [97]. Although direct evaluations of ECS modulation in these paradigms remain limited, indirect evidence supports its efficacy. FAAH inhibitors and dual FAAH/MAGL blockade reduce hyperalgesia and trigeminovascular activation in NTG-based migraine models [21,56], suggesting that endocannabinoid augmentation may counteract central sensitization similar to that observed in MOH. Additional preclinical work shows that AEA and CB1 agonists suppress nociceptive trigeminovascular activity [14], further supporting their potential to reduce the heightened excitability characteristic of MOH. On the clinical side, data indicate that some patients with refractory migraine or MOH use cannabis on their own to manage symptoms. In an observational study of patients with chronic migraine and MOH, the synthetic cannabinoid nabilone reduced headache intensity and analgesic consumption more effectively than ibuprofen [98]. A separate pilot trial suggested that nabilone decreased medication intake, improved quality of life, and reduced pain intensity in MOH patients [98]. However, these studies were limited by small sample sizes and open-label or crossover designs, underscoring the need for larger, rigorously conducted randomized controlled trials.

Despite encouraging results, several limitations hinder the translation of ECS-based therapies for MOH. First, there is a lack of robust preclinical MOH-specific studies directly testing ECS modulation. Second, cannabinoids carry risks of tolerance, dependency and paradoxical worsening of headache, especially with THC-dominant preparations [76]. Third, patient responses to cannabis are highly variable, with some individuals experiencing clinical benefits while others reporting a worsened headache [99]. Finally, strict regulations and safety concerns, including risks of dependence, cognitive effects and medication overuse, continue to limit clinical trial development.

Future research should focus on validating ECS-targeted interventions in preclinical MOH models, particularly by testing FAAH inhibitors, MAGL inhibitors, SSCIs, and CB2 agonists for their ability to prevent or reverse central sensitization associated with medication overuse. Clinically, carefully designed randomized controlled trials are needed to evaluate the efficacy and safety of ECS-targeted therapies in MOH. Biomarkers such as circulating CGRP, imaging of descending modulatory networks, and assessment of neuroimmune markers could help track therapeutic response. Peripherally biased ECS modulators and non-psychoactive cannabinoids such as CBD may provide safer alternatives for long-term management. Beyond MOH, the knowledge gained from these studies may also help in understanding and treating the wider challenges of chronic migraine and difficult-to-treat headache.

Together, the experimental and preclinical models discussed above provide complementary insights into how endocannabinoid signaling regulates headache pathophysiology across multiple levels of the nervous system. Trigeminovascular and meningeal preparations reveal direct modulation of nociceptor activity and CGRP release; cortical spreading depression models capture ECS control of cortical excitability and aura-related mechanisms; and TBI paradigms highlight the contribution of ECS modulation to persistent neuroimmune sensitization and chronic pain vulnerability. Although these models differ in structure and primary readouts, they converge on shared ECS-dependent mechanisms that shape nociceptive gain, inflammatory signaling, and descending pain control. To facilitate comparison and synthesis across these diverse experimental platforms, Table 2 summarizes the major headache models discussed in this section, highlighting their defining features, key outcome measures, and ECS-targeted interventions. This integrated overview clarifies how findings across distinct paradigms collectively inform translational strategies for ECS-based headache therapeutics.

## 4. Sex Differences in Headache and Implications for ECS-Mediated Therapies

Beyond injury-related mechanisms, biological sex profoundly influences headache vulnerability, symptom presentation, and treatment response. These differences have critical implications for ECS-mediated interventions. After puberty, migraine becomes two- to four-times more common in women than in men [100,101]. This shift mirrors the rise in estrogen fluctuations during the reproductive years. These hormonal changes strongly influence headache vulnerability and alter responsiveness to cannabinoid-based treatments. Estradiol downregulates FAAH through estrogen receptor-dependent signaling, elevating AEA and altering CB1/CB2 receptor activity [102]. These ECS fluctuations correspond to well-recognized clinical patterns, with symptoms peaking at menarche, stabilizing during pregnancy, and increasing again in perimenopause. This temporal alignment illustrates how sex hormones modulate both headache susceptibility and cannabinoid-mediated analgesic pathways [101].

At the neurobiological level, baseline ECS tone differs substantially between sexes in regions central to migraine pathophysiology. In the primary visual cortex, females exhibit distinct levels of CB1 receptors, FAAH and MAGL. This pattern points to sex-specific regulation of cortical excitability and CSD susceptibility [103]. Similar differences are found in the PAG, where CB1/CB2 expression and endocannabinoid-metabolizing enzymes vary by sex. As a result, the two sexes engage in descending pain-modulatory circuits through distinct ECS mechanisms [104,105]. These regional molecular differences translate into measurable behavioral effects. Females commonly demonstrate greater analgesic responses to cannabinoids, which may result from elevated endogenous cannabinoid tone in central nociceptive pathways. However, they also experience more cannabinoid-related adverse effects, highlighting the bidirectional influence of gonadal hormones on ECS pharmacodynamics [106].

Evidence from preclinical studies indicates that biological sex significantly influences ECS regulation of headache-related pain pathways. Because sex differences have not been systematically evaluated across all headache models, this section focuses on paradigms in which comparisons of males and females were directly performed or where sex-dependent ECS effects are most clearly documented.

Among these, the NTG-induced migraine model provides some of the strongest evidence for sex-dependent ECS modulation. Female rodents exhibit reduced AEA levels associated with elevated FAAH activity and enhanced trigeminal sensitivity compared with males, indicating a greater disruption of endocannabinoid tone in females following NTG exposure [104,107]. These findings suggest that ECS-based interventions targeting endocannabinoid metabolism may differentially influence headache susceptibility and treatment responses between sexes.

Sex differences are also documented in trigeminovascular and meningeal activation models, where females display greater mast cell activation and more pronounced microglial cytokine responses than males [108,109]. Such immune-related differences are highly relevant to ECS signaling, given its established role in regulating neurogenic inflammation within headache circuits.

Similarly, CSD models demonstrate robust sex differences in cortical excitability, with females exhibiting lower CSD thresholds and increased susceptibility compared with males [110,111]. Although these studies did not explicitly dissect ECS mechanisms by sex, they provide important context for understanding how sex-dependent differences in excitability may interact with endocannabinoid signaling in migraine with aura.

Sex-dependent ECS effects are further evident in PTH models, where females show heightened microglial and astrocytic activation and greater disruption of endocannabinoid tone following TBI, whereas males generally display less pronounced neuroimmune responses [112]. Together, these findings support a role for sex-dependent ECS regulation of neuroimmune processes that contribute to headache persistence and severity.

In contrast, sex differences in ECS modulation have not been systematically examined in TAC models, but evidence from hypothalamic ECS regulation suggests that sex-dependent mechanisms may also contribute to TAC pathophysiology.

Collectively, these studies indicate that sex differences in endocannabinoid tone, cortical excitability, and neuroimmune reactivity contribute to variability in ECS-mediated headache modulation. Recognizing these differences is essential for interpreting preclinical findings and for guiding the development of sex-informed ECS-targeted therapies for headache disorders.

These mechanistic insights are reinforced by emerging clinical evidence. Observational cohorts and real-time symptom tracking studies reveal that women often report greater therapeutic benefit from cannabis-based treatments but also experience higher rates of adverse effects, mirroring preclinical differences in ECS sensitivity and hormonal modulation [113,114]. Recent reviews indicate that cannabinoids interact with neuroimmune and trigeminovascular pathways in sex-specific ways [115]. These findings suggest that hormonal status, ECS gene expression, and metabolic differences all contribute to variation in clinical responses to cannabinoid-based therapies. Taken together, these findings highlight the need to integrate sex and hormonal factors into ECS-targeted therapy development. Prospective studies should evaluate sex differences, track the reproductive phase, and adjust dosing carefully. Combining endocannabinoid modulation with CGRP inhibition may offer particular benefit in female-dominant conditions such as migraine.

## 5. Conclusions

The endocannabinoid system functions as a multilevel regulator of headache biology, modulating cortical excitability, trigeminovascular gain, and neuroimmune activation through coordinated CB1- and CB2-mediated signaling. Across diverse preclinical models, including nitroglycerin administration, cortical spreading depression, dural stimulation, post-traumatic headache paradigms, and trigeminal autonomic cephalalgias, enhancement of endogenous AEA and 2-AG tone through FAAH, MAGL, or COX-2 inhibition consistently reduces headache-like behaviors and restores homeostatic control of pain pathways.

Sex differences exert a strong influence on these mechanisms. Females exhibit distinct endocannabinoid metabolism, heightened glial reactivity, and estrogen-sensitive CB1 signaling, contributing both to increased vulnerability to sensitization and to greater responsiveness to ECS augmentation. These biological differences parallel emerging clinical observations and underscore the importance of incorporating sex as a core variable in the development of ECS-targeted therapeutics.

Despite substantial mechanistic insights from preclinical studies, particularly in nitroglycerin-based and other migraine-relevant models, the translational impact of ECS modulation in human headache disorders remains limited. Experimental approaches used to model tension-type headache and trigeminal autonomic cephalalgias capture only selected aspects of these conditions and do not fully replicate the complexity of real attacks, making it difficult to draw firm conclusions about ECS involvement. Moreover, early clinical trials targeting ECS-related pathways have not demonstrated clear therapeutic benefit, underscoring the gap between promising preclinical findings and clinical efficacy. Taken together, these limitations highlight the need for more refined disease models, better biomarkers of ECS function, and rigorously designed clinical studies to determine whether ECS modulation can meaningfully contribute to headache treatment.

## Figures and Tables

**Table 1:** Key endocannabinoid system components relevant to headache biology.

Component	Localization in Headache-Relevant Circuits	Functional Role in Headache Pathophysiology	Therapeutic Strategy
CB1 receptor	Presynaptic terminals in cortex, PAG, RVM, TNC, trigeminal ganglion	Limits glutamate and GABA release; regulates cortical excitability, trigeminovascular gain, and descending pain control	CB1 agonists, positive allosteric modulators, peripherally biased CB1 ligands [11,13,14]
CB2 receptor	Microglia, perivascular macrophages, meningeal immune cells, peripheral immune system	Reduces microglial activation and pro-inflammatory cytokine release; attenuates central sensitization	CB2-selective agonists or biased ligands [12,15,16]
Anandamide (AEA)	Cortex, PAG, trigeminal and meningeal afferents	Acts at CB1, CB2, and TRPV1; bidirectional control of CGRP release and vascular tone; stabilizes hyperexcitable circuits	FAAH inhibition; substrate-selective COX-2 inhibition [10,13,17,18]
2-Arachidonoylglycerol (2-AG)	Cortex, PAG, RVM, TNC, trigeminal ganglion	Primary CB1/CB2 ligand; controls synaptic transmission and glial signaling; reduced in CSD and TBI-associated headache	MAGL or ABHD6 inhibition; dual hydrolase blockade [10,19,20]
FAAH	Cortex, hippocampus, meningeal tissues, trigeminal system	Degrades AEA; restricts CB1/CB2/TRPV1-mediated actions	Global or peripheral FAAH inhibitors (e.g., URB937, PF-04457845) [18,21,22]
MAGL	Cortex, PAG, TNC, perilesional brain (TBI)	Degrades 2-AG; upregulated in CSD and TBI; contributes to headache-like behaviors	Selective MAGL inhibitors; dual FAAH/MAGL inhibitors (e.g., JZL195) [23,24,25]
TRPV1/TRPA1	Trigeminal afferents, meningeal terminals, select cortical neurons	Drive CGRP release, vasodilation, and neurogenic inflammation; functionally interact with AEA	TRP antagonists or desensitizers combined with ECS augmentation [26,27,28]
COX-2 (endocannabinoid oxygenation)	Cortex, meninges, inflamed neural tissues	Converts AEA and 2-AG to prostaglandin derivatives; reduces available endocannabinoid tone	Substrate-selective COX-2 inhibitors that spare AEA/2-AG [29,30]

**Table 2:** ECS-targeted interventions across experimental and preclinical headache models.

Animal Model/Paradigm	Headache Type Modeled	Key Features/Readouts	ECS-Relevant Findings	Representative References
Trigeminovascular electrophysiology (TCC–PAG recordings)	Migraine	Dural-evoked Aδ/C-fiber firing; spontaneous TCC activity	CB1 activation suppresses trigeminovascular firing; PAG–RVM engagement	[13,14,31]
Meningeal vascular/hemiskull preparation	Migraine	CGRP release; dural blood flow	AEA induces TRPV1-dependent CGRP release; CB1 suppresses trigeminal signaling	[13,28]
Nitroglycerin (NTG)-induced migraine	Episodic & chronic migraine	Periorbital allodynia; photophobia; CGRP; cytokines	FAAH/MAGL inhibition reduces allodynia, CGRP, and inflammation	[21,22,54]
Cortical spreading depression (CSD)	Migraine with aura	CSD frequency and propagation; cortical excitability	CB1 activation and endocannabinoid preservation reduce CSD susceptibility	[20,66]
Inflammatory soup (IS) dural application	Chronic migraine-like sensitization	CGRP upregulation; TRPV1 sensitization; persistent allodynia	ECS augmentation suppresses trigeminal sensitization	[21,66]
TRPV1/TRPA1 agonist models (capsaicin, AITC)	Migraine	CGRP release; meningeal vasodilation	CB1–TRPV1 crosstalk determines net nociceptive output	[26,28]
Non-invasive dural stimulation (NIDS)	Migraine	Facial hypersensitivity; neuroinflammation	Suitable for testing peripherally restricted ECS modulators	[72]
Trigeminal autonomic cephalalgia (TAC) reflex model	Cluster headache/TAC	Lacrimation; nasal secretion; TCC firing	CB1 suppresses trigeminal–autonomic reflex activity	[74,75]
Persistent muscle pain (repeated intramuscular acid or hypertonic saline injections)	TTH (chronic)	Bilateral referred pain; pericranial muscle hyperalgesia; central sensitization; impaired descending inhibition	FAAH or MAGL inhibition restores CB1-dependent descending antinociceptive control and reduces muscle-evoked hyperalgesia	[77,78]
Stress-based pain models (repeated restraint stress; chronic mild stress)	TTH (stress-related)	Stress-induced hyperalgesia; reduced PAG–RVM inhibitory tone; enhanced central sensitization	ECS augmentation normalizes descending pain modulation and attenuates stress-evoked hypersensitivity via CB1 signaling	[79,80]
CHIMERA closed-head injury	PTH	Periorbital allodynia; glial activation; CGRP	MAGL and COX-2 substrate-selective inhibition reduces headache behaviors	[25,30]
Weight-drop TBI	PTH	Persistent cephalic allodynia; CGRP hypersensitivity	ECS implicated in long-term trigeminovascular sensitization	[85,86]
Controlled cortical impact (CCI)	PTH	Reduced AEA; microglial activation; allodynia	MAGL inhibition restores ECS tone and reduces pain	[84,88]
Fluid percussion injury (FPI)	PTH	Cortical excitability; neuroinflammation	FAAH/MAGL inhibition improves synaptic and inflammatory outcomes	[91]
Blast injury models	Military-relevant PTH	Microglial polarization; behavioral deficits	CB2 activation promotes anti-inflammatory microglial phenotypes	[92]
Medication overuse headache (triptan/opioid exposure)	MOH	Latent sensitization; enhanced nociceptive gain	ECS augmentation may counteract central sensitization	[97,98]

## Data Availability

No new data were created or analyzed in this study.

## Abbreviations

The following abbreviations are used in this manuscript:

2-AG	2-arachidonoylglycerol
ABHD6	α/β-hydrolase domain-containing protein 6
AEA	anandamide (n-arachidonoylethanolamine)
AITC	allyl isothiocyanate
BBB	blood–brain barrier
CaMKII	Ca^2+^/calmodulin-dependent protein kinase ii
CB1	cannabinoid receptor type 1
CB2	cannabinoid receptor type 2
CBD	cannabidiol
CCI	controlled cortical impact
CGRP	calcitonin gene-related peptide
CHIMERA	closed-head impact model of engineered rotational acceleration
COX-2	cyclooxygenase
CSD	cortical spreading depression
DAGLα/β	diacylglycerol lipase alpha/beta
ECS	endocannabinoid system
FAAH	fatty acid amide hydrolase
FPI	fluid percussion injury
GABA	gamma-aminobutyric acid
IL	interleukin
IS	inflammatory soup
MAGL	monoacylglycerol lipase
MAPK	mitogen-activated protein kinase
MOH	medication-overuse headache
NAPE-PLD	n-acyl-phosphatidylethanolamine–phospholipase d
nNOS	neuronal nitric oxide synthase
NF-κB	nuclear factor kappa B
NIDS	non-invasive dural stimulation
NTG	nitroglycerin
PAG	periaqueductal gray
PACAP	pituitary adenylate cyclase-activating polypeptide
PET	positron emission tomography
PTH	post-traumatic headache
RAMP1	receptor activity-modifying protein 1
RVM	rostral ventromedial medulla
5-HTT	serotonin transporter
SSCIs	substrate-selective cyclooxygenase-2 inhibitors
TAC	trigeminal autonomic cephalalgias
TBI	traumatic brain injury
TCC	trigeminocervical complex
TG	trigeminal ganglion
THC	δ^9^-tetrahydrocannabinol
TNC	trigeminal nucleus caudalis
TRPA1	transient receptor potential ankyrin 1
TRPV1	transient receptor potential vanilloid 1
VPAC1	vasoactive intestinal polypeptide type 1 receptor
vlPAG	ventrolateral periaqueductal gray
